# Tailored Anesthetic Management in a Patient With Birt-Hogg-Dubé Syndrome: Navigating the Risk of Pneumothorax and Renal Dysfunction in a Case Report

**DOI:** 10.7759/cureus.85115

**Published:** 2025-05-31

**Authors:** Nodoka Hatashima, Kan Takahashi, Junko Dozono, Hiromasa Kida, Sho Matsuba

**Affiliations:** 1 Department of Anesthesiology, Kanazawa Medical University, Ishikawa, JPN

**Keywords:** birt-hogg-dubé syndrome (bhds), multiple pulmonary cysts, pneumothorax, post renal transplant, renal dysfunction

## Abstract

Birt-Hogg-Dubé syndrome (BHDS) is a rare genetic disorder characterized by multiple pulmonary cysts, increasing the risk of pneumothorax under positive pressure ventilation. We report a case of a 50-year-old woman with BHDS, recurrent pneumothorax, and post-renal transplant chronic renal failure, who underwent three surgeries within three years. Anesthetic management was tailored to minimize the risk of pneumothorax and deterioration of renal function, including spinal anesthesia for cervical conization, combined epidural-general anesthesia with lung-protective ventilation for open sigmoid colectomy, and general anesthesia with peripheral nerve blocks for an emergency Hartmann’s procedure. Careful ventilation strategies, controlled extubation using a supraglottic airway under deep anesthesia, and renal-protective management employing regional anesthesia successfully prevented perioperative complications. This case highlights the need for individualized anesthetic management in BHDS patients with renal transplants, emphasizing careful strategies to protect both pulmonary and renal function.

## Introduction

Birt-Hogg-Dubé syndrome (BHDS) is a rare autosomal dominant disorder caused by mutations in the FLCN gene, which encodes folliculin, a tumor suppressor protein involved in cellular energy regulation and mTOR (mammalian target of rapamycin) signaling pathways [1]. Clinically, BHDS is characterized by a triad of benign skin tumors (fibrofolliculomas), multiple pulmonary cysts, and an increased risk of renal neoplasms [1,2]. From an anesthesiology standpoint, BHDS presents a unique set of perioperative challenges. Pulmonary cysts increase the risk of spontaneous pneumothorax, particularly during procedures that require general anesthesia with positive pressure ventilation [3]. Consequently, anesthesiologists often favor regional anesthesia techniques - such as spinal or epidural anesthesia - to reduce pulmonary barotrauma, maintain spontaneous ventilation, and preserve lung integrity. However, BHDS may also involve renal complications requiring surgical intervention, such as nephrectomy for renal tumors and subsequent kidney transplantation. In post-transplant patients, the anesthetic plan must also account for preservation of renal function. This dual-system vulnerability - pulmonary and renal - significantly narrows the margin for safe anesthetic management and necessitates a tailored, multidisciplinary approach to perioperative care.

In this case report, we describe the perioperative management of a patient with BHDS and post-transplant renal dysfunction, who underwent three different surgeries: cervical conization, open sigmoid colectomy, and an emergency Hartmann’s procedure - managed with spinal anesthesia, combined epidural and general anesthesia, and general anesthesia with a peripheral nerve block, respectively. All procedures were successfully completed without complications. The objective of this report is to highlight practical anesthetic strategies that address both pulmonary protection and renal preservation in BHDS patients [4]. Through this case, we aim to contribute to a broader understanding of individualized, risk-adapted anesthetic planning in patients with multisystemic genetic disorders, thereby enhancing perioperative safety and outcomes in this rare but clinically important population.

## Case presentation

**Figure 1:**
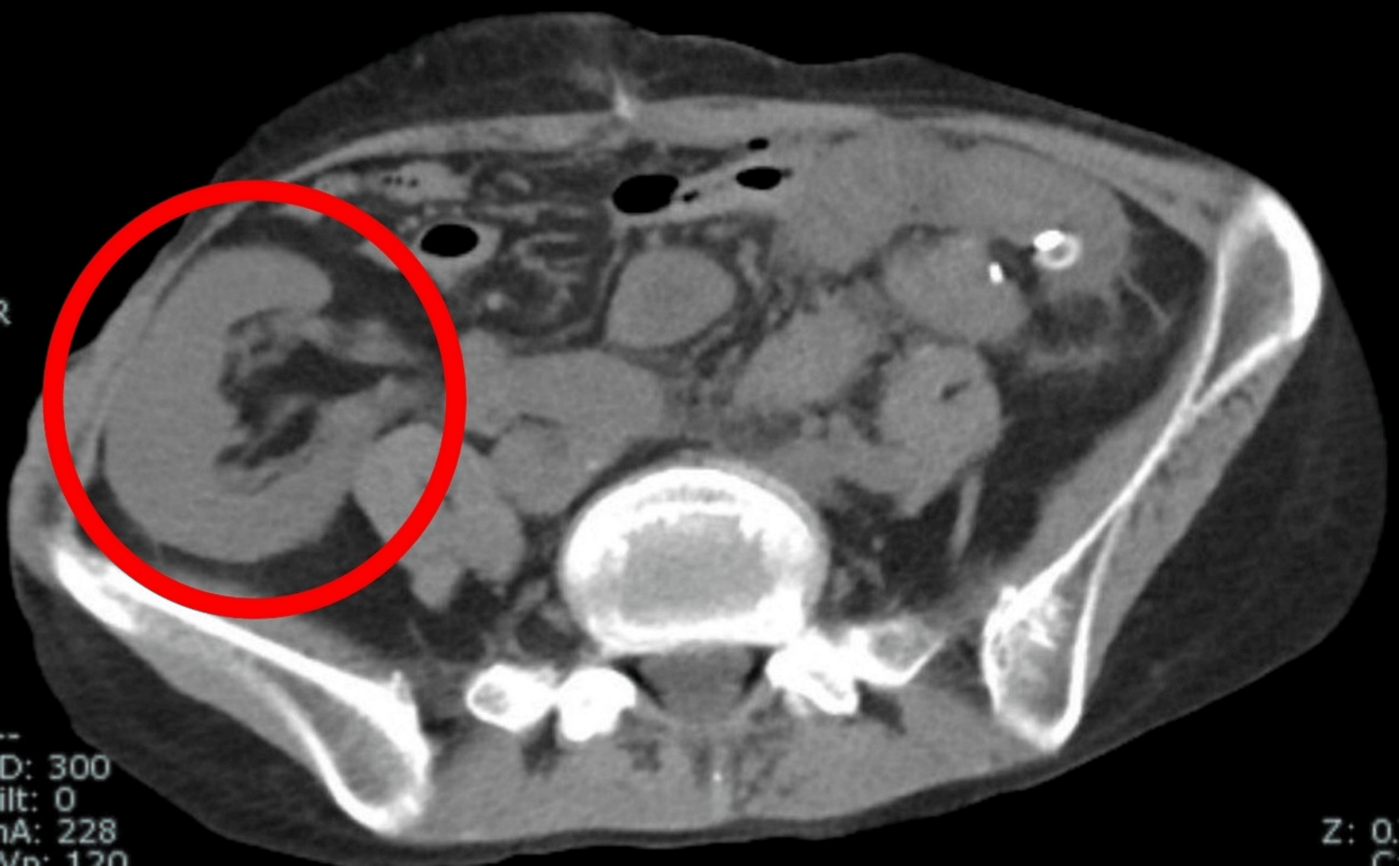
Preoperative abdominal CT (axial) showing transplanted kidney. Abdominal CT showing a transplanted kidney in the right iliac crest (red circle). CT, Computed Tomography

A 50-year-old woman (height 156 cm and weight 44 kg) experienced her first spontaneous pneumothorax at the age of 15, followed by recurrent episodes requiring surgery at the ages of 17 (open bulla resection), 29, and 35 (thoracoscopic bulla resection). Renal dysfunction was noted at the age of 15, progressing to hemodialysis by the age of 24 due to chronic glomerulonephritis. A living-donor kidney transplant was performed at the age of 25, with her mother as the donor (Figure 1). At the age of 30, she underwent right nephrectomy for renal cell carcinoma. Multiple facial and cervical papules appeared at the age of 34 and were treated dermatologically. She had a notable family history of pneumothorax, with five of her seven paternal siblings similarly affected. One sibling was diagnosed with BHDS, and subsequent genetic testing confirmed the same diagnosis in the patient at the age of 35.

Since the renal transplant, the patient had been continuously taking steroids (methylprednisolone 6 mg/day) and immunosuppressants (azathioprine 50 mg/day). Facial lesions later developed, and, at age 36, given her family history of pneumothorax, she was diagnosed with BHDS through genetic testing. She was also undergoing medical treatment for mixed anxiety-depressive disorder.

First surgery

**Figure 2:**
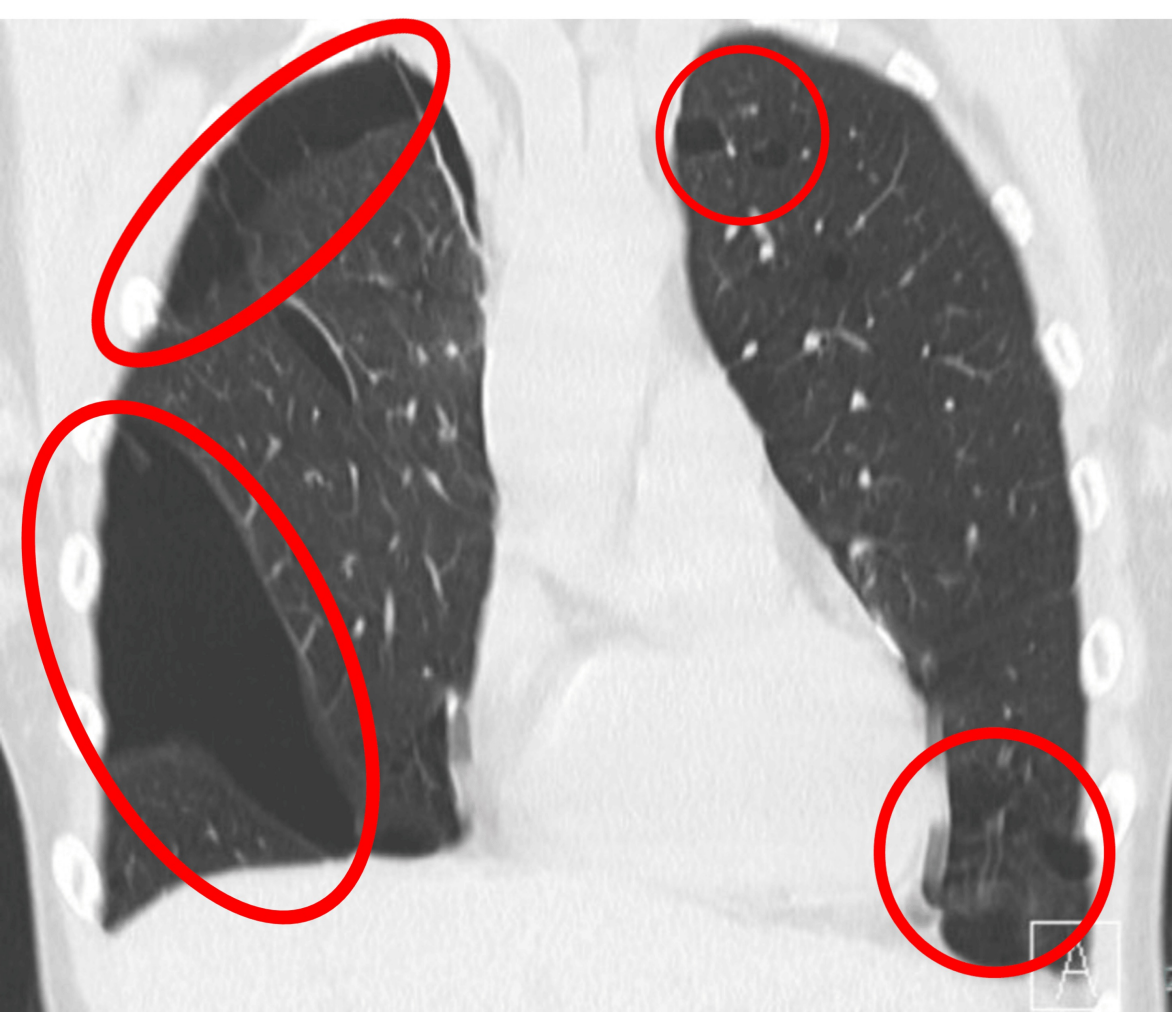
Preoperative chest CT (coronal) showing multiple pulmonary cysts. A chest CT shows numerous bilateral pulmonary cysts (red circles and ovals). CT, Computed Tomography

**Figure 3:**
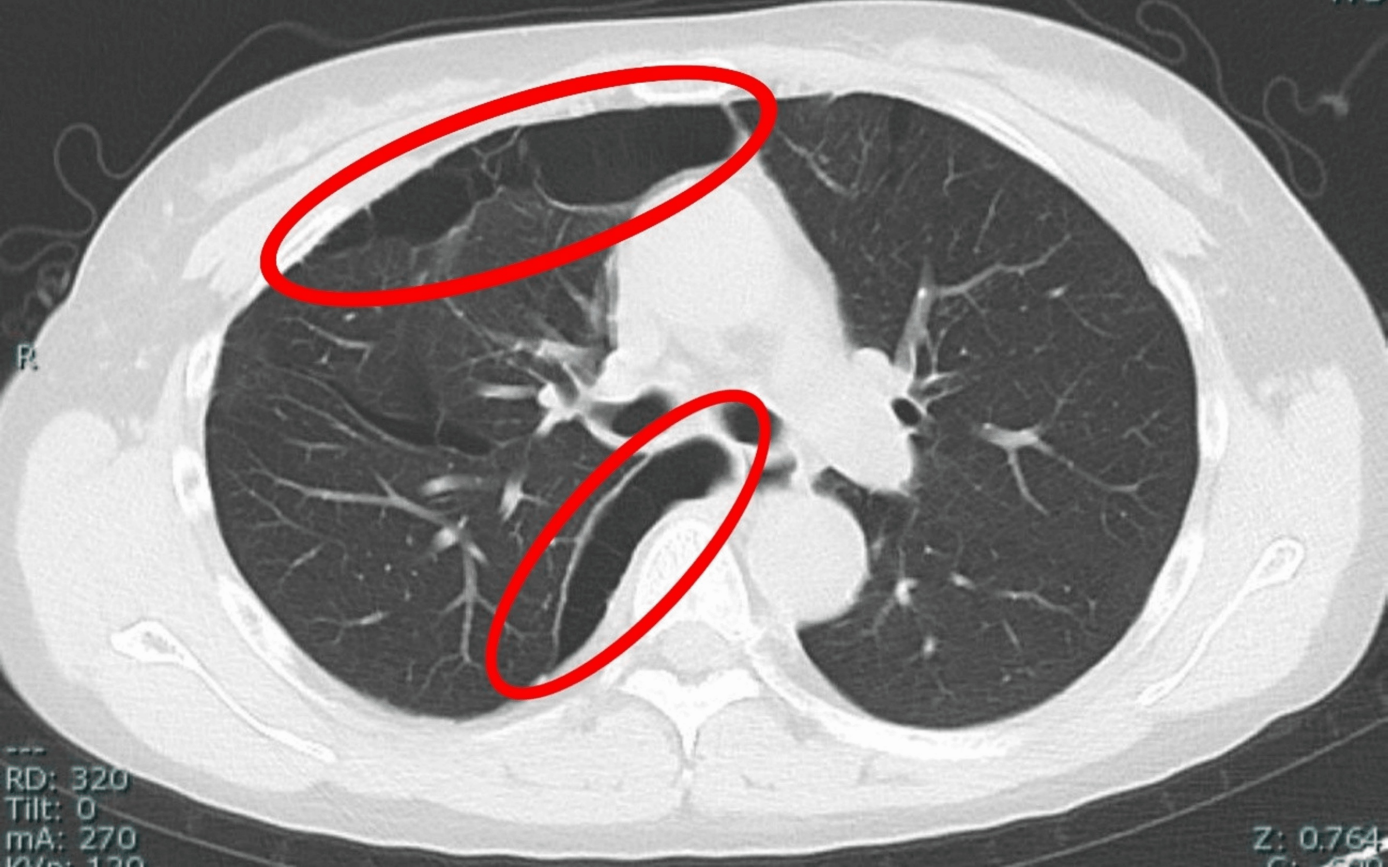
Preoperative chest CT (axial) showing multiple pulmonary cysts. Multiple pulmonary cysts are observed, predominantly in the right middle and lower lung fields, and along the mediastinal side (red ovals). CT, Computed Tomography

Cervical conization for cervical intraepithelial neoplasia was planned. Preoperative computed tomography (CT) revealed numerous bilateral pulmonary cysts, predominantly in the middle and lower lung fields, and along the mediastinal side (Figures 2-3). Spinal anesthesia was chosen to avoid the risk of pneumothorax from positive pressure ventilation. Isobaric 0.5% bupivacaine, 3 mL, was administered at the L2/3 level, achieving a sensory block below T7. The procedure was uneventful, with no signs of postoperative respiratory distress or pneumothorax.

Second surgery

The second surgery was scheduled three years after the first procedure for open sigmoid colectomy to treat sigmoid colon cancer. Blood tests revealed creatinine levels of 2-2.5 mg/dL and an eGFR of 20-25 mL/min/1.73 m², indicating impaired renal function. The patient had adequate spontaneous urination, with no electrolyte abnormalities or elevated blood urea nitrogen (BUN). CT revealed pulmonary findings similar to those of the first surgery. A combination of epidural and general anesthesia was selected, considering the patient’s mental state and surgical requirements. After performing epidural anesthesia at the Th10/11 level, rapid induction was achieved using propofol and rocuronium. Anesthesia was maintained with sevoflurane, fentanyl, and 1% lidocaine administered through the epidural catheter. Pressure-controlled ventilation was set with an inspiratory pressure of 10 cm H₂O, a positive end-expiratory pressure (PEEP) of 4 cm H₂O, and a respiratory rate of 14 breaths per minute, resulting in a tidal volume of 300-320 mL. No signs of pneumothorax or respiratory compromise were observed intraoperatively or postoperatively. Postoperative renal function deterioration was not observed.

Third surgery

**Figure 4:**
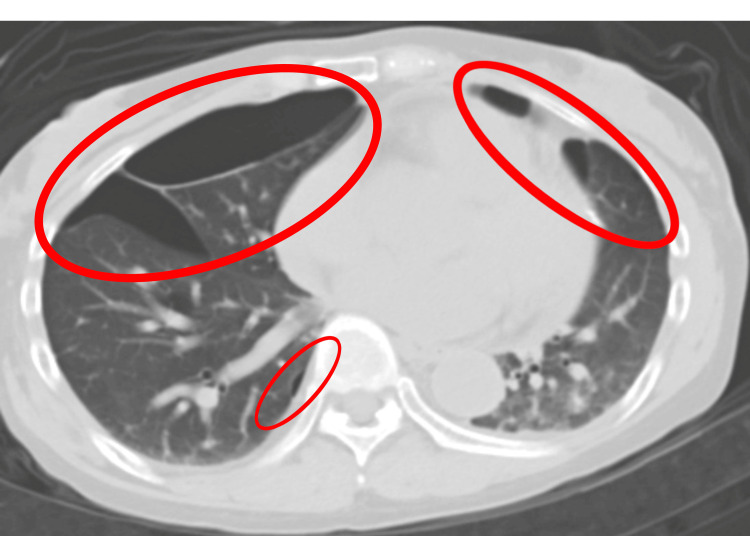
Postoperative chest CT (axial) showing no pneumothorax after third surgery. Postoperative chest CT revealing pulmonary cysts in the same locations (red ovals) as preoperatively, indicating no pneumothorax. CT, Computed Tomography

One month later, we performed an emergency Hartmann’s procedure for colonic anastomotic leakage. Given the risk of sepsis, epidural anesthesia was avoided, and general anesthesia with a peripheral nerve block was used. Rapid induction was performed with propofol and rocuronium, and anesthesia was maintained with sevoflurane, fentanyl, and remifentanil. The ventilator settings were adjusted similarly to those used during the second surgery. No signs of tension pneumothorax were observed intraoperatively. Pain management included fentanyl, acetaminophen, and a rectus sheath block with 0.25% levobupivacaine. Ventilator settings similar to those in the second surgery were used. Extubation was carefully managed by exchanging the endotracheal tube for an i-gel® laryngeal mask (Intersurgical Ltd., Wokingham, UK) to minimize airway pressure. No pneumothorax occurred during or after the procedure (Figure 4). The clinical courses of the three surgical procedures are summarized in Table 1.

**Table 1:** Clinical courses of the three surgical procedures. We summarized the preoperative data, anesthesia methods, intraoperative vital signs, postoperative analgesia, and postoperative course. Cr, Creatinine; eGFR, Estimated Glomerular Filtration Rate; IV-PCA, Intravenous Patient-Controlled Analgesia; PIP, Peak Inspiratory Pressure; PEEP, Positive End-Expiratory Pressure; RR, Respiratory Rate; CT, Computed Tomography; BP, Blood Pressure

Parameter	First Surgery	Second Surgery	Third Surgery
Surgical Procedure	Cervical conization	Open sigmoid colectomy	Emergency Hartmann’s procedure
Indication	Cervical intraepithelial neoplasia	Sigmoid colon cancer	Colonic anastomotic leakage
Pulmonary CT Findings	Multiple bilateral lung cysts	Same as the first surgery	Same as the second surgery
Pre-operative Renal Function	Cr 1.7 mg/dL, eGFR 20-25 mL/min/1.73 m^2^	Cr 2.2 mg/dL, eGFR 20-25 mL/min/1.73 m^2^	Cr 2.0 mg/dL, eGFR 17-22 mL/min/1.73 m^2^
Post-operative Renal Function (POD3)	Cr 1.81 mg/dL, eGFR 24.7 mL/min/1.73 m^2^	Cr 1.78 mg/dL, eGFR 24.7 mL/min/1.73 m^2^	Cr 2.3 mg/dL, eGFR 18.6 mL/min/1.73 m^2^
Anesthetic Technique	Spinal anesthesia	Combined general and epidural anesthesia	General anesthesia + rectus sheath block
General Anesthetics	Not used	Propofol, rocuronium, sevoflurane, fentanyl	Propofol, rocuronium, sevoflurane, remifentanil
Ventilation Mode	Spontaneous breathing	Pressure-controlled ventilation	Pressure-controlled ventilation
Ventilator Settings	Not applicable	PIP 10 cm H₂O, PEEP 4 cm H₂O, RR 14/min	Same as the second surgery
Pneumothorax	None	None	None
Mean BP Throughout the Surgery	88 mmHg	66.4 mmHg	57.4 mmHg
Intraoperative Fluid Volume	300 mL/hr	450 mL/hr	600 mL/hr
Urine Output	Not measured	30 mL/hr	10 mL/hr
Postoperative Course	Uneventful	Uneventful	Uneventful
Postoperative Analgesia	Not specified	Epidural ropivacaine	Rectus sheath block, IV-PCA (fentanyl)

## Discussion

An estimated 663 families with the disorder are reported globally. BHDS patients may also present with other benign or malignant tumors, such as thyroid, breast, and colorectal tumors [5,6]. The selection of anesthetic techniques must be tailored to the tumor type, surgical approach, and patient condition to minimize perioperative complications. To our knowledge, this is the first report of a BHDS patient with renal dysfunction after kidney transplant, requiring multiple general anesthetics within such a short period. 

More than 80% of BHDS patients develop multiple pulmonary cysts, making them highly susceptible to pneumothorax due to positive pressure ventilation. Several reports have described successful anesthetic management using regional techniques to avoid positive pressure ventilation, including cases of prostatectomy managed with combined spinal-epidural anesthesia, and laparoscopic cholecystectomy under thoracic segmental spinal anesthesia, which demonstrated uneventful outcomes without pneumothorax [7].

A previous study reported that undiagnosed BHDS patients undergoing surgeries under general anesthesia may experience postoperative pneumothorax [8]. The study reported a case of abdominal wall reconstruction and liposuction, where airway pressures of 22-26 mmHg during a three-hour surgery contributed to pneumothorax. Similarly, a case of laparoscopic partial nephrectomy resulted in postoperative pneumothorax when managed with tidal volumes of 12-15 mL/kg and PEEP of 5 cm H₂O [9]. The high compliance of lung cysts, compared with normal lung tissue, predisposes these structures to overdistension and rupture under excessive airway pressures [10].

In managing this patient, we applied pressure-controlled ventilation mode, ensuring that airway pressures did not exceed 15 cm H₂O. Ventilator management based on previous reports enabled us to prevent overdistension of pulmonary cysts and subsequent pneumothorax [11]. Beyond ventilation strategies, postoperative extubation also requires careful attention to avoid struggling and coughing, which can suddenly increase intrathoracic pressure and potentially trigger pneumothorax. For the second surgery, combined epidural anesthesia facilitated pain management and reduced the risk of straining during extubation. For the third surgery, epidural anesthesia was avoided due to potential sepsis risks; instead, we employed regional blocks and intravenous patient-controlled analgesia (IV-PCA) with fentanyl for postoperative analgesia, and extubation was performed under deep anesthesia, with an i-gel® supraglottic airway in place to mitigate airway pressure spikes. Similar strategies have been reported in thoracic surgery cases involving large pulmonary cysts, where exchanging a double-lumen tube for an i-gel® prior to extubation minimized airway pressure and reduced the risk of cyst rupture [12].

Despite thorough airway management, pneumothorax remains a potential risk in patients with multiple pulmonary cysts. General anesthesia with positive pressure ventilation should be avoided as much as possible. It is essential to ensure clear communication among anesthesiologists, surgeons, and operating room staff regarding this risk. Preparedness to rapidly diagnose and treat pneumothorax - including ensuring the availability of chest ultrasound, portable chest X-rays, and chest drain insertion equipment - is crucial for optimal safety for BHDS patients.

Although the relationship with BHDS remains unclear, the patient underwent living-donor kidney transplantation at the age of 25 due to chronic glomerulonephritis. Throughout the perioperative management period, careful attention is required for pulmonary cysts and impaired renal function. Approximately 20% of patients with BHDS develop renal tumors, typically occurring after their 40s or 50s [13]. Renal tumors in BHDS are often treatable with surgical resection, and cases requiring kidney transplantation are rare [14]. In the present case, the patient had undergone living-donor kidney transplantation but exhibited chronic renal failure, with a creatinine level of about 2 mg/dL and an eGFR of about 20 mL/min/1.73 m². By minimizing the use of intravenously administered anesthetics that are metabolized by the kidneys, employing regional anesthesia, and actively maintaining blood pressure to preserve renal blood flow, we were able to prevent deterioration of renal function.

## Conclusions

The present case illustrates the complexity of perioperative management in patients with BHDS, particularly those with a history of renal transplantation. Given the risks associated with pulmonary cysts and renal dysfunction, anesthetic plans should be individualized, with a focus on minimizing pulmonary barotrauma and preserving renal function. This report emphasizes the importance of interdisciplinary collaboration and vigilant perioperative care in ensuring favorable outcomes in such multifaceted clinical scenarios.
